# NK Cells as Possible Prognostic Factor in Childhood Acute Lymphoblastic Leukemia

**DOI:** 10.1155/2019/3596983

**Published:** 2019-01-02

**Authors:** Agnieszka Mizia-Malarz, Grażyna Sobol-Milejska

**Affiliations:** Department of Pediatrics, School of Medicine in Katowice, Medical University of Silesia, Upper Silesia Children's Care Health Centre, Katowice, 16 Medykow Street, Poland

## Abstract

Deficiency or impaired function natural killer (NK) cells might result in the development of serious infections and promote the development of malignancies. The aim of our study was to assess the prognostic role of NK cell percentage in bone marrow on the day of acute lymphoblastic leukemia (ALL) diagnosis. 84 children (49 males = 58%; median age 5 yrs) with ALL were enrolled. The NK cell percentage was assessed using flow cytometry with antibodies against the cluster of differentiation (CD): CD3, CD56, and CD16. We evaluated two groups: group I (NK+), patients with NK cells in the bone marrow (*n* = 74), and group II (NK-), patients without NK cells in the bone marrow (*n* = 10) (cut-off value of negative <1%). In the patients from group I, the prednisone good response on day 8 and the remission on day 15 of treatment were observed significantly more often (*p* = .01, *p* = .03). The children from group I had significantly better survival as compared to those from group II (*p* = .02) (HR 2.59; 95% CI: 1.38-4.85). The presence of NK cells in the bone marrow at diagnosis can be a prognostic factor in children with ALL. The presented results should be the basis for further research.

## 1. Introduction

Acute lymphoblastic leukemia (ALL) is the most common malignancy in children [1, 2]. ALL treatment outcomes in children tend to gradually improve over time. Currently, the 5-year survival is over 80% and mainly depends on the risk factors assessed upon diagnosis and at early stages of treatment [3–5]. According to the Acute Lymphoblastic Leukemia Intercontinental 2002 (ALL-IC 2002) treatment protocol, the prognostic factors in ALL children include patient age, white blood cell (WBC) count at baseline, cytogenetic abnormalities (presence of t(9,22) or t(4,11) chromosome abnormalities), and early treatment response markers [6]. New prognostic factors emerge along with the increasing knowledge of ALL. According to the current treatment protocol (Acute Lymphoblastic Leukemia Intercontinental 2009; ALL-IC 2009), hypodiploid blasts at the diagnosis and minimal residual disease (MRD) assessed by flow cytometry on day 15 of treatment are considered new crucial prognostic factors in ALL children, along with the previously identified ones [7]. Further research will likely yield new prognostic factors.

NK cells are the immune system components. Their role in both eliminating infectious agents and destroying malignant cells has been discussed [4–13]. Considering constant advances in the identification of novel prognostic factors and improving treatment outcomes in pediatric malignancies, such as ALL, the authors attempted to address the question whether, due to their natural function, NK cells could become a new prognostic factor in ALL children.

The purpose of our research was to assess the prognostic role of NK cell presence in bone marrow evaluated on the day of ALL diagnosis in affected children.

## 2. Material and Methods

A total of 84 newly diagnosed children with ALL (median age 5 years; range 3–10; gender M/F 49(58%)/35(42%) were diagnosed in the Department of Pediatrics, Medical University of Silesia, Upper Silesian Child Health Care Centre in Katowice, Poland, between 2005 and 2013.

The diagnosis was based on morphological, cytochemical, immunophenotypic, cytogenetic, and molecular bone marrow analyses. Immunophenotyping was performed using flow cytometry. The following monoclonal antibodies were included in the immunophenotyping panel: cluster of differentiation (CD) 2, cyt CD3, CD3, CD5, CD7, CD10, CD19, CD20, CD34, CD117, CD13, CD14, CD15, CD16, CD33, CD45, CD56, CD66, MPO, and HLA-DR. The antigen expression rate ≥ 20% was considered significant.

All study participants received treatment in line with the Acute Lymphoblastic Leukemia Intercontinental 2002 Protocol (ALL IC 2002) (*n* = 47; 56%) and Acute Lymphoblastic Leukemia Intercontinental 2009 Protocol (ALL IC 2009) (*n* = 37; 44%) and were accordingly classified into appropriate risk groups: standard-risk group (SRG) *n* = 24 (28.6%), intermediate-risk group (IRG) *n* = 46 (54.8%), and high-risk group (HRG) *n* = 14 (16.6%). The presence of t(9,22) chromosome abnormality in leukemic cells was detected in 5 (6%) children. We have not detected genetic abnormalities such as ETV6/RUNX1, MLL/AF4, and TCF3/PBX1. The response to the treatment, according to the protocol, on day 8 was assessed on lymphoblast count in the peripheral blood and on days 15 and 33 based on the lymphoblast percentage in the bone marrow. For the assessment on day 8, prednisone good response (PGR) was defined as <1000 blasts/*μ*l and prednisone poor response (PPR) was defined as ≥1000 blasts/*μ*l. For the assessment of bone marrow on days 15 and 33, the following grading scale was used: M1, lymphoblast count <5%; M2, lymphoblast count ≥5 and <25%; M3, lymphoblast count ≥25%. In the children treated according to ALL-IC 2009 protocol (*n* = 37), minimal residual disease (MRD) was additionally assessed by flow cytometry on day 15 of treatment, with MRD >10% considered an eligibility threshold for the high-risk group (Table 1).

The NK cell percentage in the bone marrow at the day of the diagnosis was assessed using flow cytometry with monoclonal antibodies against CD3, CD16, and CD56.

All participants were divided into 2 groups: group I (NK+), patients with NK cells (≥1%) in the bone marrow *n* = 74 (88%), including 67 patients with precursor B cell ALL (BPC ALL) and 7 patients with T ALL, and group II (NK-), patients without NK cells (<1%) in the bone marrow *n* = 10 (11%), including 8 patients with BPC ALL and 2 patients with T ALL (Table 1).

Patient's parents agreed to the necessary examinations during routine diagnostic procedure at the day of diagnosis ALL. We used the results of these examinations in this work.

Statistical analysis was performed using MedCalc Statistical Software version 13.1.0 (MedCalc Software bvba, Ostend, Belgium; http://www.medcalc.org; 2014). All of the text and table results are expressed as mean ± standard deviation (SD) or median and IQR or number and percentage. The normal distribution of the results was analyzed using the D'Agostino-Pearson test. Baseline clinical parameters and the results of accessory investigations were compared using the *t*-test or Mann-Whitney *U* test. Categorical variables were compared using the Chi-squared or Fisher's exact test. Survival analysis of patients with and without NK cells was made using the Kaplan-Meier method. Cox regression was used to analyze the effect of several risk factors on the survival. The results were expressed with 95% confidence interval (95% CI). Results of *p* < .05 were considered statistically significant.

## 3. Results

In the patients from group I, a good response to steroid therapy (PGR) was observed significantly more often on day 8 of treatment (*p* = .01) (Table 1). Similarly, the remission on day 15 of treatment was observed significantly more often in this group (*p* = .03) (Table 1).

There were no significant differences between groups I and II as to risk groups (SRG, IRG, and HRG), treatment protocol, and response to the treatment assessed on day 33 (Table 1).

The mean follow-up time from the end of treatment was 1516 days (25-75Q: 990-2823). 71 patients (84.5%) survived, whereas 13 patients (15.5%), including 9 from group II, died.

The analysis demonstrated significantly better survival in children from group I as compared to those from group II (*p* = .02) (HR 2.59; 95% CI: 1.38 – 4.85).

## 4. Discussion

There is a continuous pursuit to improve the outcomes of pediatric cancer treatment, including leukemia. Therefore, new prognostic factors are identified, which offer treatment optimisation. The role of natural immune components, including NK cells, in fighting cancer has been studied [4, 8, 9, 11–13].

NK cells are derived from common lymphoid progenitors (CLPs) and undergo a pro-NK stage, after which they differentiate into pre-NK cells and subsequently into NK cells [14]. They are characterised by the expression of CD16, CD56, and CD57 antigens, with the absence of CD3. NK cells do not need the presence of major histocompatibility complex (MHC) on target cells due to direct lytic action of perforins, granzymes, cytokines, and interferon gamma (IFN*γ*), which they release, on target cells [8, 9]. The decreased MHC expression is observed in virus-infected cells and malignant cells. These cells become a direct target for the NK cells [8, 15, 16]. It was demonstrated that the deficiency or impaired cytotoxic function of NK cells might result in the development of serious infections and promote the development of malignancies [5, 10–12, 15, 16].

Frishman-Levy et al. [1] demonstrated the ability of NK cells to directly destroy blasts in ALL. NK activation is triggered by interleukins, e.g., IL-15, the serum level of which is elevated in patients with leukemias. We believe that the above NK cell function is of great importance as it may translate into improved control of systemic peripheral leukemia. However, it has no impact on the treatment outcomes in ALL patients with central nervous system (CNS) involvement, since according to Frishman-Levy et al. [1] NK cells are excluded from the CNS. This is why in our opinion direct prevention of CNS involvement based on IL-15-activated autologous NK cells should be considered [1, 17, 18].

According to Boieri et al. [18], NK cell therapies can offer hope for better treatment outcomes in leukemia. The authors proved significantly lower proliferation leukemic cells in the presence of NK cells, especially activated by Il-12, Il-15, and Il-18 [18]. Also according to Jin et al. [19], interleukin-activated NK cells cause the elevation of IFN gamma levels, which additionally enhances the antineoplastic effect.

A number of authors discussed the role of NK cells in graft versus leukemia (GvL) effect in patients after hematopoietic stem cell transplantation (HSCT) [1, 4, 9, 20–22]. In children with high risk ALL (HR ALL) after haploidentical HSCT alloreactive NK cells have been shown to contribute to improved treatment outcomes through the GvL effect [4, 9, 20, 21, 23–26].

According to Torelli et al. [4], ALL blasts in children are characterised by increased surface expression of ligands triggering NK cell activation. As a result, NK cells from healthy bone marrow donors may significantly contribute to ALL blast destruction, in particular in BCR/ABL (+) patients, thus preventing ALL recurrence [4, 26]. Therefore, we believe that the future use of NK cell therapy in ALL should be considered, especially in BCR/ABL (+) patients.

The literature review indicates a particularly important role of NK cells in neoplastic disease. Owing to their properties, NK cells can offer hope for better treatment outcomes in children with ALL. Our results appear to confirm the beneficial effect of NK cells on treatment outcomes in children with ALL. According to the current ALL treatment protocol followed in Poland (ALL-IC 2009), prednisone response on day 8 and MRD on day 15 of treatment are crucial prognostic factors. Good responders at those time points tested positive for NK cell presence upon diagnosis. Additionally, the presence of NK cells upon diagnosis was a positive prognostic factor for survival in the studied group of children.

Just as Frishman-Levy et al. [1] and Kubler et al. [9], we would like to echo the questions, whether it will be possible to include NK cells in the future ALL treatment protocols and whether it will help reduce multidrug chemotherapy administered as a standard of care as per current treatment protocols. Further research on NK cells in leukemia may help address these questions in the future.

The presence of NK cells in the bone marrow upon diagnosis can be an independent prognostic factor in children with acute lymphoblastic leukemia. The presented results should be the basis for further research.

## Figures and Tables

**Table 1:** Characteristics at initial diagnosis of group I (NK+) and group II (NK-).

Variables	Overall (*N* = 84)	Group		*p* value
		Group I (NK+) (*N* = 74) median (25-75Q) *N* (%)	Group II (NK-) (*N* = 10) median (25-75Q) *N* (%)	
BCP ALL/T ALL Chi-squared test p 0.16	75/9 (89.3/10.7%)	67/7 (90.5/9.5%)	8/2 (80/20%)	
Age (yrs) median (range)	5 (3–9)	5 (3–10)	5 (4–8)	0.91
Gender (M/F)	49/35 (58/42%)	42/32 (56.8/43.2%)	7/3 (70/30%)	0.02
Ph(+)/Ph(-)	5/79 (6/94%)	4/71 (5/95%)	1/8 (11/89%)	0.95
Protocol ALLIC 2002/2009	47/37 (56/44%)	40/34 (54/46%)	7/3 (70/30%)	0.29
Risk groups SRG/IRG/HRG	24/46/14 (28.6/54.8/16.6%)	22/40/12 (29.8/54/16.2%)	2/6/2 (20/60/20%)	0.84
Day 8 PGR/PPR	78/6 (92.8/7.2%)	71/3 (96/4%)	7/3 (70/30%)	0.01
Day 15 bone marrow M1/M2/M3	63/13/8 (75/15.5/9.5%)	60/7/7 (81/9.5/9.5%)	3/6/1 (30/60/10%)	0.03
Day 15^∗^ (*N* = 37) MRD <0.01/0.01-≤10.0/>10.0	18/11/8 (48.6/29.8/21.6%)	16/10/8 (47.1/29.4/23.5%)	2/1/0 (66.7/33.3/0%)	0.03
Day 33 bone marrow M1/M2 + M3	80/4 (95/5%)	70/4 (94.6/5.4%)	9/1 (90/10%)	0.81

Bone marrow M1 < 5% lymphoblasts; bone marrow M2 ≥ 5–<25% lymphoblasts; bone marrow M3 ≥ 25% lymphoblasts. Day 15: minimal residual disease was made in 37 pts. HRG: high-risk group. IRG: intermediate-risk group. SRG: standard-risk group. MRD: minimal residual disease. Ph (+): Philadelphia chromosome positive. Ph (-): Philadelphia chromosome negative. PGR: prednisone good response (blast cells in peripheral blood < 1000.0/*μ*l). PPR: prednisone poor response (blast cells in peripheral blood ≥ 1000.0/*μ*l).

## Data Availability

Data from children with leukemia used in this study were collected in the excel database and are in the author's of this work hands. The results of the statistical analysis of these data were placed on this manuscript.
